# Exploring Knowledge and Experience of Health Literacy for Chinese-Speaking Nurses in Taiwan: A Cross-Sectional Study

**DOI:** 10.3390/ijerph17207609

**Published:** 2020-10-19

**Authors:** Ya-Wen Chang, Tsai-Chung Li, Yen-Chin Chen, Jo-Hua Lee, Mei-Chuan Chang, Li-Chi Huang

**Affiliations:** 1 School of Nursing, China Medical University, Taichung 406040, Taiwan; yawen172@mail.cmu.edu.tw; 2 Department of Public Health, China Medical University, Taichung 406040, Taiwan; tcli@mail.cmu.edu.tw (T.-C.L.); N55302@mail.cmuhch.org.tw (Y.-C.C.); 3 Department of Emergency, China Medical University Hsinchu Hospital, Hsinchu 302, Taiwan; 4 Department of Nursing, Asia University Hospital, Taichung 41354, Taiwan; johua.lee@gmail.com; 5 Department of Nursing, Tzu Chi University, Hualien 970, Taiwan; meichuan2@gmail.com; 6 Department of Nursing, China Medical University Hospital, Taichung 40447, Taiwan

**Keywords:** nursing, health literacy, patient education, nurses’ knowledge of health literacy, nurses’ experience of health literacy

## Abstract

*Background*: Health literacy has become the best predictor of healthcare status. However, two-thirds of health providers are unaware of patients’ health literacy. Thus, the aim of the study is to investigate factors related to Chinese-speaking nurses’ knowledge and experience of health literacy. *Methods*: This cross-sectional study used a web-based survey. A total of 430 nurses were recruited by stratified sampling from different levels of hospitals and community health centers in Taiwan. Primary outcome measure by Health Literacy Knowledge and Experience. *Results*: The participants’ overall health literacy knowledge was limited; the correct responses were 51%. The education level of the participants, job category, working years, and having attended in-service patient education programs were the predictors of knowledge of health literacy (*p* < 0.05); Institute, job category, and having attended in-service patient education programs were the predictors of experience of health literacy (*p* < 0.01). *Conclusions*: Participants’ education levels and In-service patient education programs are beneficial factors to improve nurses’ knowledge of health literacy. Furthermore, nursing education should emphasize on how to identify individuals’ health literacy and using readable healthcare materials to improve health education.

## 1. Introduction

Health literacy is a rapidly growing issue in health care, and it has become the best predictor of healthcare status [1]. Health literacy is defined as competency related to the process of accessing, understanding, appraising, and applying health information in order to make decisions in everyday life [2]. One of the nurse’s roles is as an educator, providing health information to improve the individual’s health literacy for health promotion and disease management [3,4]. Thus, assessing and addressing health literacy is a priority in the provision of quality patient education [5]. Nurses’ knowledge and experience of health literacy is, therefore, important for health provision.

Low health literacy is a recognized global problem [2,6,7]. In Taiwan, approximately 30% of adults have low health literacy [8]. People with low health literacy appear to have difficulty reading and understanding basic health information [2,9], lower rates of medication compliance [10], a higher rate of hospitalization [8], less health promotion behavior [11,12], and 1.5–3 times more likely to have poor health status [13]. Related studies estimate that the extra annual cost of low health literacy compared with that of persons with adequate health literacy range from the US $ 143–7798 per person, per year [14]. Low caregiver health literacy is associated with both a higher rate of prior emergency department visits and greater odds of a non-urgent emergency department visit and health outcomes [15,16]. Studies indicated people with low health literacy tended to be older, less educated and from lower socioeconomic groups [2,6,8]. People with low health literacy were usually poor at communication and less likely to ask questions [17]. Unfortunately, healthcare professionals may not recognize these characteristics of low health literacy and may not be aware of their impact on health outcomes [18].

A lack of knowledge among the providers of health literacy-related issues can substantially affect patient-provider communication and hinder the benefits of health care [19,20]. It has been shown that two-thirds of health providers are unaware of their patients’ health literacy and the lack of policies about health literacy [21]. One study showed that 63% of the patients had a likelihood of limited health literacy, while nurses reported 19% of patients having a likelihood of limited health literacy [21]. Studies also indicate that health providers commonly overestimate patients’ literacy [21,22]. Even more than 80% of nurse practitioners never or rarely formally assess health literacy via a validated screening tool [19]. This misunderstanding of patients’ health literacy may prove to be an important barrier to health provision.

Modern health systems are complex and it is difficult to understand health-related information, especially for people with low literacy; and even people who are well-educated [6,21]. It has been recommended that all written materials should be tested for readability to ensure they are suitable for the health literacy level of patients before patient education [23,24]. The Joint Commission on Accreditation of Health Organizations has recommended the reading level for patient education materials should ideally be equivalent to those of school grade 5 or lower [25]. Currently, many tools available are for evaluating health literacy and written materials. The Rapid Estimate of Adult Literacy in Medicine (REALM) and the Test of Functional Health Literacy in Adult (TOFHLA) are widely used to assess an individual’s health literacy level [6,26]. The Fry readability formula is a way of determining the readability of material [27]. Several tools have also been developed for the Chinese. The short-form Mandarin Health Literacy Scale (s-MHLS) and The Taiwan Health Literacy Scale (THLS) are simple tools to assess Chinese-speaking peoples’ ability to read, comprehend and use basic health information [28,29]. There are web-based systems and the Chinese Readability Index Explorer (CRIE) for analyzing the readability of Chinese written materials [30]. The Suitability Assessment of Materials (SAM) is the tool that most commonly used to assess the adequacy of written healthcare materials, and it has been translated into Chinese [31].

Many researchers have concluded that health literacy not only improves the abilities of individuals but that it also removes barriers to patient understanding of the health system [6,23]. As first-line care providers, nurses’ knowledge and experience of health literacy are vital to patients’ health promotion. Several studies have examined knowledge of health literacy among nursing students [3,32], nurse practitioners [19,33], and fewer studies in nurses [34], but none of these studies explore the phenomenon in Chinese-speaking nurses. These studies found the health literacy knowledge of nurses or nursing students to below. However, the gaps in content between the studies and the factors related to knowledge of health literacy have not yet been established. Hence, the purpose of this study was to investigate the knowledge and experiences of nurses of health literacy and related factors.

## 2. Materials and Methods

### 2.1. Study Design

A descriptive, cross-sectional, web-based survey through Google sheets was used to assess the characteristics associated with knowledge and experience of the health literacy of Chinese-speaking Registered Nurses.

### 2.2. Participants and Settings

We used different sampling approaches for participants from the hospital and community. Taiwan hospitals are distributed into district hospitals, regional hospitals, and medical centers with different functions. The district hospitals are responsible for general inpatient and specialist outpatient care, and continuous medical work, with the number of beds ranging from 20–99; Regional hospitals play a leading role in the community medical system which involve teaching and training functions, with more than 300 beds in general; The medical center has a variety of functions such as research, teaching, and care of acute and critical patients, with more than 500 beds [35]. Most hospitals request nurses with bachelor’s degrees. The roles of nurses in the community health center are community care providers and family health managers.

For hospitals, stratified sampling was conducted based on the type of hospital, with a medical center, a regional hospital, two district hospitals being selected by convenience sampling method. Under each selected hospital, simple random sampling was used to select wards and all nurses of selected wards were recruited, which is cluster sampling. In the selected medical center and regional hospital, wards were stratified by the type of ward (medical and surgical ward). Participants from community health centers were stratified by urbanization level (urban and rural areas). Rural areas were further stratified into the mountain and seaside districts while urban areas were further stratified into districts. Under each area, convenience sampling was used to select health centers. All the nurses of the selected centers were recruited, which is cluster sampling. Nurses in Taiwan have three-month probation before officially working clinically. Thus, only nurses who had worked for at least three months were recruited. Nurses who were not currently working with patients in the hospital and community were excluded. The sample size was estimated by using multiple regression with Omnibus (R^2^ deviation from zero) in G power V3.0.10 software (University of Dusseldorf, Dusseldorf, Germany). Effect size f_2_ = 0.07 was converted from R^2^ = 0.07 of pilot study (*n* = 30). Thus, considering the number of potential predictors (*n* = 10) in this study, the effect size of f_2_ = 0.07 (small) with α = 0.05 to detect the association between knowledge and personal characteristics, the sample size of 358 would reach a power of 95%. An additional 72 subjects were added to accommodate that 20% of subjects might lose, resulting in a total of 430 participants being recruited. With a final sample size of 400, the study had a power of 97% to yield a statistically significant result.

### 2.3. Tool

#### 2.3.1. Translation and Modification of the Tool 

The tool used to assess participants’ knowledge and experience of health literacy was translated and modified from Health Literacy Knowledge and Experience Survey (HL-KES), which was developed by Cormier and Kotrlik [32]. The HL-KES has been used to measure the knowledge and experience of health literacy in nursing students [3,32] and nurse practitioners [19,33], but not in Chinese-speaking nurses. Thus, we translated and adapted this into Chinese by the WHO process, which includes: (1) The forward translation was by one health professional who was familiar with health literacy terminology. The stage aims to make the conceptual equivalent of sentences and avoid using jargon; (2) Expert panel: two experts identified and resolved the inadequate expressions of the translation; (3) Back-translation to English was made by a translator, whose mother language is English and has no knowledge of health literacy. This stage of translation emphasized conceptual and cultural equivalence and not linguistic equivalence; (4) Pre-testing with 30 nurses, recruited by convenience sampling from outside of formal study hospitals, and cognitive interviewing was also conducted to test the participants’ understanding and accessibility [36]. We modified aspects of the survey related to cultural ethnicity and screening tools. For example, the original item, Low health literacy levels are common among: (a) African Americans; (b) Hispanic Americans; (c) White Americans; (d) All ethnic groups. We changed the responses in current ethnicities in Taiwan to: (a) Native Taiwanese; (b) New immigrants; (c) Fujianese; (d) Hakka; (e) All ethnic groups.

Although REALM was translated into Chinese, the use of language and text is fundamentally different from Chinese and thus cannot be applied directly [29]. The THLS, a local health literacy scale was based on the Chinese health vocabulary of the National Health Service in Taiwan [29]. Therefore, HL-KES item #10 on REALM was replaced by THLS. The s-MHLS scale was used to test health-related reading and comprehension skills. The question is designed around four health care scenarios, including actual health information, visits, medication guidelines, and medical service documents. Thus, the s-MHLS replaced the original item #13 of Functional Health Literacy. Item #21, the Fry Method was also changed to readability analysis in the Chinese system. Moreover, SAM has been extensively used to evaluate the suitability of healthcare materials. It has been translated into Chinese [31]. Therefore, it is included in item #30.

This instrument comprising 48 items was divided into three subscales. The first subscale consisted of seven items about demographic data including age, gender, education level, working years, job category, institutions, and in-service patient education programs. The second subscales consisted of 30 multiple-choice questions regarding knowledge of health literacy, including basics facts of health literacy, low health literacy, health literacy screening, methods of written healthcare materials, and evaluation of interventions. The correct answer scores 1, while incorrect answer scores 0, and total scores ranged from 0–30. The third subscale focused on participants’ experience of health literacy, including core health literacy and technology health literacy experience [32]. Eleven items were scored on a four-point Likert scale from ‘never’ (1) to ‘always’ (4). Scores ranged from 11–44, higher scores indicated better knowledge and experience of health literacy. In addition, two open-ended questions were included to obtain a better understanding of the participants’ opinions: 1. ‘What was the greatest difficulty you experienced during your health education?’ 2. ‘What would you suggest to improve current health education?’ The entire survey took approximately 15–20 min to complete.

#### 2.3.2. Validity and Reliability of Instrument

Content validity was determined by a panel of five experts who were: a nurse supervisor and a senior nurse in health literacy practice, a health educator who was health literacy training, a physician who developed the health literacy scale, and a nursing professor who had health literacy grant from Taiwan’s Health Promotion Administration. For the purpose of the study, these experts reviewed the appropriateness, clarity, and vocabulary of the content and then revealed a CVI value of 0.98 [37]. The face validity of the knowledge and experience of health literacy was conducted by a pilot test of 10 nurses (from outside of formal study hospitals). The internal consistency reliability of the HL-KES was resulted using a KR-20 value of 0.69 in knowledge, and Cronbach’s alpha 0.76 in the experience.

### 2.4. Data Collection and Analysis

The study was approved by the Institutional Review Board at a university hospital (CRREC-106-081). The researcher obtained cooperation from the nurse director in each hospital and community health center. The researcher explained the purpose of the study to the participants in the ward meeting or during the shift. At the same time, the researcher provided the printed QR code card with a self-administered online questionnaire, which could be used on a smartphone or a PC. The aims and purpose of the study were provided on the front page of the survey system. After reading the information, participants clicked to agree to participate in the study and started to answer the questionnaire. The data were collected from 1 December 2017–28 February 2018.

The statistical analysis was performed using SPSS Statistics version 22 (IBM, Armonk, NY, USA). Descriptive statistics of frequency and proportion for categorical variables and mean with standard deviation (SD) for continuous or ordinal variables were used to describe the distributions of variables. We used inductive qualitative content analysis to analyze responses to the two open-ended questions. An independent *t*-test was used to compare the means of two groups while ANOVA with the posthoc Bonferroni test was used for multiple group comparisons. Spearman’s correlation coefficients were used to analyze the relationship between the continuous or ordinal type of demographic data and knowledge and experience of health literacy. Multiple generalized linear modeling was used to analyze the associations between explanatory variables and outcomes [38]. For explanatory variables, we use the variance inflation factor (VIF) to perform the collinearity diagnosis. In general, a VIF value over 10 is a clear signal of multicollinearity. The Durbin-Watson statistic approaching two (2) means less autocorrelation [39]. Statistical significance was designated at an alpha level of 0.05.

## 3. Results

### 3.1. Demographic Data

After checking for duplicates and incomplete surveys, 400 participants completed the study (a completion rate of 93%). Of these 391 (98%) were female with mean age 32.86 (SD = 9.28) years, range 20–65 years. Mean work experience was 10.30 (SD = 8.95) years, range 0.3–40 years. Most (73.0%; *n* = 291) had a bachelor’s degree, 76.8% were clinical nurses and 35% had attended in-service patient education programs. There were 91.5% of participants from hospital-based (39.8% medical center, 34.2% regional hospital, 17.5% district hospital) while 8.5% of participants from the community center (Table 1).

The question “what was the greatest difficulty you experienced during your health education?”, answered by 232 participants, indicated: communication barriers between nurses and individuals (48%); patients and families with insufficient motivation (34%); and limited time, space and education materials for health education (24%). The question “what would you suggest to improve current health education?”, answered by 85 participants, indicated developing easy to understand and more illustration materials (62%); providing teaching space and equipment (22%); and improving nurses’ health education skills (18%).

### 3.2. Knowledge and Experience of Health Literacy

Table 2 shows the participants’ correct responses to the knowledge of health literacy. The mean correct response rate was 51%, range 19–82% (Table 1). The mean score for knowledge of health literacy was 15.35 (SD = 4.12), range 3–25. Item #22: “Recommendations for developing written healthcare materials should present information in the conversational form” was most commonly scored correct (82%). and item #19: “The recommended reading level for written healthcare information is 5th grade” was scored correct least (19%). Item #11: “People with low health literacy may not admit that they have difficulty reading” was answered correctly by only 27%. 

Only 20% responded correctly that the THLS was used for assessing patients’ health literacy levels, 38% that the s-MHLS was used for assessing patients’ health literacy levels, 23% that the readability analysis of Chinese system, and 38% that the SAM was used for assessing the suitability of health materials.

The mean score for core health literacy experience and technology health literacy experience was 25.90 (SD = 7.08), range 11–44 (Table 3). Fifty-nine percent of the participants frequently or always used written materials. More than half of the participants answered that they ‘never’ or ‘sometimes’ used screening tools to assess individuals’ health literacy or to evaluate the level of written healthcare materials. The experiences in using technology showed that 70% of participants never or sometimes used audiotapes, 60% of participants never or sometimes used videotapes and computer software and demonstrate.

For the purpose of the study, the related factors of nurses’ knowledge and experience of health literacy were examined. The *t*-test and ANOVA were used to exam the difference between the variables. The participants’ age, education level, working years, job category, health institutes, and in-service patient education programs all showed statistically associations with knowledge of health literacy (*p* < 0.05; Table 1). The participants’ job category, institutes, and in-service patient education programs were statistically associated with their experience of health literacy (*p* < 0.05; Table 1). The response variables were tested the normal distribution and results as non-normal distribution. Thus, a generalized linear model (GLM) was used for the purpose of the study. All the above significant variables Table 1 was tested by the VIF index for individual knowledge and experience of health literacy. VIF of job category and institutes were both larger than 10. Therefore, we excluded the variable of institutes in the multiple GLM analysis. The participants’ education level, the working year, job category, and in-service patient education programs were predictors of knowledge of health literacy (*p* < 0.05; Table 4); and job category, and in-service patient education programs were the predictors of experience of health literacy (*p* < 0.05; Table 5) after adjusting for other variables in the generalized linear models. The DW statistics in the models of Table 4 and Table 5 were 1.86 and 1.63, respectively.

## 4. Discussion

### 4.1. Knowledge of Health Literacy

To our knowledge, this is the first study to investigate the nurses’ knowledge and experience of health literacy and factors in Chinese-speaking nurses. Our study showed that nurses’ knowledge of health literacy is limited. They responded correctly to only 51% of the items on knowledge of health literacy, a much lower result than those of other studies in Western countries [33,34]. In this study, approximately one-fifth of participants know that the best reading level for written health materials is 5th grade. Most participants misbelieve that the best reading level is grades 9 to 12. This finding is consistent with results in other studies [3,32]. The Joint Commission on Accreditation of Health Organizations (2010) highly recommended that education materials should be written at the 5th- grade reading level or below, as measured by readability tools, to address the health literacy needs of all patients [25]. Even people with a high level of education may still have problems understanding medical information [21]. Although, many studies have reported that health professionals were not aware of the issues associated with a high reading level of printed materials and low literacy of the individual [21,22,40], which acts as a major barrier to patients that wish to understand health information.

Our study finds that most of the participants are not aware of available health literacy screening tools, similar to other previous studies, 80% reported that they never or rarely assessed health literacy using a validated tool; and 60% responded that they used their gut feeling to estimate a patient’s health literacy level [3,19,32]. Scholars in Taiwan have developed instruments for assessing patients’ health literacy (such as s-MHLS, THLS, and MMHLQ) [28,29,41], the readability (such as Chinese Readability Index Explorer) [30], and suitability of printed materials (such as SAM in Chinese version) [31], which may account for the lack of awareness. Assessing and addressing the health literacy of individuals and selecting appropriate health education materials have not been emphasized. We suggest that health literacy competency be strengthened in nurses’ education. However, scholars in this area have argued about how the results of the testing tools in written health materials should be interpreted [40]. We should recognize that healthcare materials are tools for transmitting information, whose main purpose is to supplement rather than to replace the nurses’ role of health education [42]. Health professionals are educators who improve patients’ health knowledge based on their characteristics and cultural context [43]. Indeed, with the aid of good health materials, health professionals could improve the extent to which they convey healthcare knowledge. A diabetes nurse educator used the concept of health literacy for assessment and identified that the issue of the main caregiver is lacking of functional and interactive health literacy, and use easy to read instructions, plain language to success improved the communication and insulin injection skill [44]. Obviously, if the nurses have knowledge of health literacy and use relevant assessment tools and health education instructions, it will promote the disease management.

We also show that most participants erroneously believed that people with low health literacy would frequently ask questions. This result is very different from other studies [32,33]. In reality, these people often do not admit that they have difficulty reading, and they usually respond by saying they will take the material home to read or that they do not wear glasses [45]. Researchers have pointed out that most patients with reading problems are ashamed, and noncompliant [45]. They usually hide their inability to read and never appear to need help; some never even talk to their families about this [5,43]. People with low health literacy usually experience worse communication with their care providers and are less likely to ask questions. Thus, the most prudent approach should take universal precautions that presume low health literacy for all individuals accessing health care [46].

### 4.2. Experience of Health Literacy

Two third of the participants ‘never’ or ‘sometimes’ used technology, such as multimedia instructional materials, videos, computer software, smartphone Apps, and models. Approximately 60% of the participants frequently or always used written materials, but there was a disparity in assessing the appropriateness of materials with illustration and culture. In answering one of the open-ended questions, the participants suggest that easy-to-understand materials with more illustrations and visual videos are needed for enhancing patients’ understanding of health information. Scholars suggest that health educators be aware of the diversity of instructional tools and their characteristics and find out which are available for learners; this would make health education more interesting and more effective [9,43]. We suggest that Registered Nurses be prioritized to become the health literacy trainees of developing health literacy educational materials given to the nature of their work as well as expertise. Moreover, The Institute of Medicine (IOM) has proposed ten attributes of health literacy that health care organizations should apply to the design and distribution of print, audio-visual and social media content to ensure it is easy to understand for their target audience to understand [47]. Thus, developing various health materials with easy-to-read and illustration are needed, especially those with low literacy. Both nurses and health organizations should pay attention to it.

### 4.3. Predictors of Health Literacy Knowledge and Experience

Participant’s working years, education level, job category, and in-service patient education program were found as predictors of knowledge of health literacy. The longer the working year the better knowledge of health literacy was found in this study. With years of working experience, people would gain the lessons and effectiveness of working. An article published in The Lancet [48] found that every 10% increase in the proportion of nurses with a bachelor’s degree was associated with a 7% decrease of mortality in hospitals. In our study, nurses with higher education levels had higher knowledge of health literacy. Increasing nurses’ educational level is a current focus of education policy in Taiwan [49]. Although 84% of participants in this study had obtained a bachelor degree, awareness of knowledge of health literacy in clinical practice was low. Participants with in-service patient education had lower knowledge of health literacy in this study. Maybe more specific health literacy education would provide better training and learning in nursing practice. A study of health education with Diabetics patients’ health literacy would provide nurses to use assessment tools and health instructions and benefit patients’ disease management [44]. Job category, health educators, and community nurses’ knowledge of health literacy are better than clinical nurses. Schlichting’s study (2007) showed that community health center providers used more health education techniques and materials to help patients [46]. Health educators and community nurses would have more training in health education and practicing in patients’ health promotion.

Participant’s job category and in-service patient education programs were the predictors of experience of health literacy. Participants who are nursing practitioners (NPs) have less experience in health literacy than clinical nurses. NPs in Taiwan commenced hospital in 1984 to cope with the shortage of physicians and to reduce costs [50]. The NPs practice all in hospitals and certification were implemented not until 2008 [51]. Fan’s study showed that Taiwanese NPs were still seen as assistants to doctors [51]. Perhaps it is the reason for less patient health education in the Taiwanese NPs’ working category.

Nearly half of the participants had never received training in patient education. The participants who had undergone patient education programs had better scores of experience of health literacy but had low scores of knowledge of health literacy. This finding could indicate that participants believed they had patient education training but still lacked sufficient knowledge in health literacy. Nursing education at all levels should place greater emphasis on nurses’ health teaching skills based on an individual’s health literacy and providing appropriate health materials in nursing practice [9,19,20,52]. The American Nurses Credentialing Centre (2009) have declared their goal of increasing nurses’ health literacy awareness in continuing nursing education, thereby improving patients’ education [5]. Moreover, organizational policies for health promotion can encourage nurses to improve their knowledge and competencies [53].

### 4.4. Practice Implications

We highlight that nurses should be aware that individuals with low health literacy may not ask questions about what they do not understand. Health providers should keep in mind that it is not possible to assume anyone’s health literacy. We suggest that nurses use the s-MHLS, THLS, or MMHLQ health literacy screening tools as part of the health education process for recognizing low health literacy in Chinese individuals. Nurses should also be aware of the features of low health literacy and use questions such as: ‘What do you know/Can you tell me about what I just told you?’ instead of: ‘Do you understand?’, because yes-or-no questions may not receive an accurate answer if the individual is embarrassed to admit that they do not understand. In particular, the ‘teach-back’ technique should be used to verify the patient’s comprehension and understand patients’ readability [52,53]. Another critical step for patient education is to apply the SAM guidelines and the Chinese Readability Index Explorer tool for selecting appropriate health materials.

### 4.5. Limitations

This study had several limitations. First, the use of a self-administered questionnaire may have resulted in a reporting bias related to social desirability. Second, all the participants were from the central district of Taiwan; the findings may not be generalizable to the Registered Nurse population throughout Taiwan. Third, the cultural difference could be a bias in this group of participants, Asian participants have high cooperation behavior that might affect the results of the study. Finally, a large regional or national survey would help to further assess registered nurses’ knowledge of health literacy in health education.

## 5. Conclusions

This study has provided evidence as a reference for the promotion of health literacy. We conclude that the participants’ education level and in-service patient education programs are beneficial factors that affect knowledge of health literacy. Nurses are at the forefront of patient education. It is important for nurses to assess an individual’s ability to understand, process, and apply health information. Thus, we suggest that school and continuing education should include health literacy in the curriculum to promote nurses’ knowledge and skills related to health literacy. Many countries have promoted health literacy as one of the main policies for public health. In 2018, Taiwan’s Ministry of Health and Welfare (MOHW) began the establishment of a health literacy resource integration center. Many programs, conferences, and workshops have been put on by city governments, academia, and health institutes [54]. We suggest longitudinal studies will highlight the trend for training priorities in nursing education and this may lead to the growth of health literacy in the health industry.

## Figures and Tables

**Table 1:** Distribution of nurses’ knowledge and experience of health literacy (*N* = 400).

Variable		N (%)	Knowledge					Experience			
			Mean	SD	*p*-Value	Bonferroni		Mean	SD	*p*-Value	Bonferroni
Gender ^a^											
	Male	9 (2.2)	15.67	5.48	0.81			25.56	6.78	0.88	
	Female	391 (97.8)	15.34	4.09				25.91	7.09		
Age ^b^											
	1. under 25	116 (29.0)	15.12	4.39	0.007 **	5 > 2		25.73	6.36	0.71	
	2. 26–30	78 (19.5)	14.01	4.48				25.15	6.94		
	3. 31–35	64 (16.0)	15.80	3.39				25.72	7.76		
	4. 36–40	65 (16.3)	15.82	3.57				26.31	6.52		
	5. over 41	77 (19.3)	16.27	4.03				26.70	8.12		
Education ^b^											
	1. College and under	85 (21.2)	13.96	3.78	*p* < 0.001 ***	3 > 1,2		26.59	7.28	0.11	
	2. Bachelor	291(72.8)	15.51	4.14				25.46	6.89		
	3. Master	24 (6)	18.33	3.05				28.71	8.10		
Working years ^b^											
	1. Under 1	28 (7.0)	14.75	4.90	0.004 **	4 > 3		25.96	7.36	0.66	
	2. 1–5	128 (32.0)	14.99	4.48				25.27	6.34		
	3. 6–10	89 (22.3)	14.46	3.79				26.01	7.13		
	4. Over 11	155 (38.8)	16.26	3.68				26.34	7.59		
Job category ^b^											
	1. Clinical nurse	307 (76.8)	14.88	2.19	*p* < 0.001 ***	5 > 1		26.02	6.94	0.04 *	5 > 2
	2. Nurse practitioner	19 (4.8)	16.21	3.24				21.37	6.66		
	3. Health educator	7 (1.8)	19.29	2.93				29.43	5.44		
	4. Case management	13 (3.3)	16.23	3.52				24.23	7.64		
	5. Community nurse	36 (9.0)	17.50	3.23				27.17	7.30		
	6. other	18 (4.5)	16.00	4.03				25.83	8.16		
Institution ^b^											
	1. Medical center	159 (39.8)	15.33	4.11	0.01 **	4 > 1,2,3		24.36	6.22	*p* < 0.001 ***	2 > 1,3
	2. Regional hospital	137 (34.2)	15.06	4.19				28.14	7.34		
	3. District hospital	70 (17.5)	14.89	4.09				24.19	7.04		
	4. Community health center	34 (8.5)	17.56	3.31				27.56	7.33		
In-service patient education programs ^a^											
	1. N	261 (65.2)	15.69	3.99	0.02 *			24.58	6.78	*p* < 0.001 ***	
	2. Y	139 (34.8)	14.71	4.29				28.37	6.99		

Note: *—*p* < 0.05; **—*p* < 0.01; ***—*p* < 0.001; ^a^—independent *t*-test; ^b^—ANOVA.

**Table 2:** Participants correct responses of knowledge of health literacy (*N* = 400).

Item	Correct Responses	
	*n*	%
Basic Facts on Health Literacy		
1. Low health literacy levels are most prevalent among which of the following age groups?	246	61.5
2. Low health literacy levels are common among:	244	61.0
3. The research on health literacy indicates that:	138	34.5
4. What is the likelihood that a nurse working in a public health clinic, primarily serving low- income minority patients, will encounter a patient with low health literacy skills?	90	22.5
5. The best predictor of healthcare status is:	210	52.5
17. An individual with functional health literacy will be able to:	282	70.5
Consequences Associated with Low Health Literacy		
6. Patients with low health literacy skills:	298	74.5
7. Health behaviors common among patients with low health literacy skills include:	134	33.5
8. Patients cope with low health literacy skills by:	159	39.8
9. The nurse should keep in mind that individuals with low health literacy levels:	277	69.3
Health Literacy Screening		
10. The Taiwan Health Literacy Scale is an instrument utilized to:	80	20.0
11. When working with individuals who have low health literacy skills the nurse should keep in mind that these individuals:	110	27.5
12. Which of the following questions would provide the nurse with the best estimate of reading skills of the patient?	219	54.8
13. Which statement best describes the short-form Mandarin Health Literacy Scale? This instrument is:	153	38.3
14. What is the strongest advantage to conducting health literacy screenings? Health literacy screenings:	302	75.5
15. Which of the following statements, made by the nurse, would be the best approach to initiating a health literacy screening with a patient?	319	79.8
Guidelines for Written Healthcare Materials		
18. Which of the following is true with regards to written healthcare information?	192	48.0
19. The recommended reading level for written healthcare information is:	75	18.8
20. The first step in developing written healthcare information is to:	299	74.8
21. Which of the following statements best describes the analysis in Chinese version?	94	23.5
22. Recommendations for developing written healthcare materials include:	329	82.3
23. When listing side effects for a handout on chemotherapy the oncology nurse should limit the list to:	230	57.5
24. Written healthcare information provided to a patient related to a specific disease should include:	286	71.5
25. Which of the following would be the most effective wording for a heading in a brochure on hypertension?	170	42.5
26. The best way to ensure that a breast cancer prevention brochure is culturally appropriate is to:	217	54.3
27. Which of the following instructions on the management of diabetes would be best understood by an individual with low health literacy skills?	257	64.3
28. Which of the following approaches to patient education provides minimal opportunity for the patient to actively engage in learning?	216	54.0
30. Suitability assessment of material is a tool and can be used as the following:	152	38.0
Evaluation of Health Literacy Intervention		
16. After providing written healthcare information to a patient he states, “Let me take this information home to read.” This may be a clue to the nurse that the patient:	102	25.5
29. The most effective way for a nurse to determine how well a patient with low health literacy skills understands healthcare information is to:	248	62.0
**Average Correct Rate**	**51.1**	

**Table 3:** The distribution of nurses’ experience of health literacy (*N* = 400).

Item	*n* (%)				Mean (SD)
	Never	Sometimes	Frequently	Always	
Core Health Literacy Experience (CHLE)					
1. How frequently was health literacy emphasized in your nursing curriculum?	45 (11.3)	124 (31.0)	162 (40.5)	69 (17.3)	2.64 (0.90)
2. How often did you use a health literacy screening tool to assess the health literacy skills of an individual?	94 (23.5)	142 (35.5)	137 (34.3)	27 (6.8)	2.24 (0.89)
3. How often did you evaluate the reading level of written healthcare materials before using them for patient teaching?	63 (15.8)	164 (41.0)	141 (35.3)	32 (8.0)	2.36 (0.84)
4. How often did you evaluate the cultural appropriateness of healthcare materials, including written handouts, videos, audiotapes, before using them for patient teaching?	47 (11.8)	152 (38.0)	162 (40.5)	39 (9.8)	2.48 (0.83)
5. How often did you evaluate the use of illustrations in written healthcare materials before using them for patient teaching?	40 (10.0)	144 (36.0)	171 (42.8)	45 (11.3)	2.55 (0.82)
6. How often did you use written materials to provide healthcare information to an individual or community group?	31 (7.8)	132 (33.0)	168 (42.0)	69 (17.3)	2.69 (0.85)
Technology Health Literacy Experiences (THLE)					
7. How often did you use audiotapes to provide healthcare information to an individual or community group?	111 (27.8)	170 (42.5)	94 (23.5)	25 (6.3)	2.08 (0.87)
8. How often did you use videotapes to provide healthcare information to an individual or community group?	75 (18.8)	166 (41.5)	129 (32.2)	30 (7.5)	2.29 (0.86)
9. How often did you use computer software to provide healthcare information to an individual or community group?	85 (21.3)	155 (38.8)	128 (32.0)	32 (8.0)	2.27 (0.89)
10. How often did you use smartphone APP to provide healthcare information to an individual or community group?	128 (32.0)	146 (36.5)	108 (27.0)	18 (4.5)	2.04 (0.88)
11. How often did you use model to demonstrate healthcare information to an individual or community group?	88 (22.0)	153 (38.3)	124 (31.0)	35 (8.8)	2.27 (0.90)
Total					25.90 (7.08)

**Table 4:** The generalized linear model of knowledge of health literacy for the demographic factors (*N* = 400).

Characteristics		β	SE	*t*	*p*	95% CI
Intercept		17.12	1.07	16.07	<0.001 ***	(15.03–19.22)
Age						
	Under 25 (reference)					
	26–30	−1.08	0.74	−1.45	0.15	(−2.54–0.39)
	31–35	−0.27	0.98	−0.27	0.79	(−2.19–1.66)
	36–40	−0.71	1.08	−0.66	0.51	(−2.84–1.42)
	Over 41	−1.13	1.16	−0.97	0.33	(−3.42–1.16)
Education						
	Bachelor (reference)					
	Master	1.93	0.93	2.08	0.04 *	(0.10–3.75)
	College and under	−1.62	0.50	−3.23	0.001 **	(−2.61–0.64)
Working years						
	Over 11 (reference)					
	6–10	−1.77	0.74	−2.39	0.02 *	(−3.24–0.31)
	1–5	−1.28	0.97	−1.31	0.19	(−3.18–0.63)
	Under 1	−1.70	1.25	−1.36	0.17	(−4.08–0.52)
Job category						
	Clinical nurse (reference)					
	Nurse practitioner	0.58	0.98	0.59	0.56	(−1.36–2.51)
	Education nurse	3.90	1.55	2.52	0.01 **	(0.85–6.94)
	Case management	1.27	1.13	1.12	0.26	(−0.96–3.50)
	Community nurse	1.73	0.79	2.18	0.03 *	(0.17–3.29)
	other	0.11	1.02	0.11	0.91	(−1.89–2.11)
In-service patient education program						
	No (reference)					
	Yes	−0.97	0.42	−2.3	0.02 *	(−1.79–0.14)

Note: Dependent Variable: knowledge; CI—Confidence Interval; *—*p* < 0.05; **—*p* < 0.01; ***—*p* < 0.001.

**Table 5:** The generalized linear model of experience of health literacy for the demographic factors (*N* = 400).

Characteristics		β	SE	t	*p*	95% CI
Intercept		24.74	0.46	53.24	<0.001 ***	(23.82–25.65)
Job category						
	Clinical nurse (reference)					
	Nurse practitioner	−3.95	1.62	−2.44	0.02 *	(−7.12–0.77)
	Education nurse	2.60	2.61	1.00	0.32	(−2.52–7.73)
	Case management	−2.47	1.93	−1.28	0.20	(−6.27–1.33)
	Community nurse	1.01	1.20	0.84	0.40	(−1.35–3.37)
	Other	0.49	1.66	0.29	0.77	(−2.77–3.74)
In-service patient education program						
	No (reference)					
	Yes	3.65	0.72	5.04	<0.001 ***	(2.23–5.07)

Note: Dependent Variable: experience; CI = Confidence Interval; *—*p* < 0.05; ***—*p* < 0.001.
